# Natural Salicylates and Their Roles in Human Health

**DOI:** 10.3390/ijms21239049

**Published:** 2020-11-28

**Authors:** Fatema Yeasmin, Hyong Woo Choi

**Affiliations:** Department of Plant Medicals, Andong National University, Andong 36729, Korea; fatema.setudu@gmail.com

**Keywords:** salicylates, pharmacological role, amorfrutin, ginkgolic acid, grifolic acid, tetrahydrocannabinolic acid, cannabidiolic acid

## Abstract

Salicylic acid (SA) is a plant hormone which plays a crucial role in the plant defense against various pathogens and abiotic stresses. Increasing reports suggest that this phenolic compound and its derivatives, collectively termed salicylates, not only regulate plant defense but also have beneficial effects on human health. Both natural and synthetic salicylates are known to have multiple targets in humans, thereby exhibiting various appreciating pharmacological roles, including anti-inflammatory, anticancer, neuroprotective, antidiabetic effects, and so on. The role of some salicylates, such as acetylsalicylic acid (aspirin), 5-aminosalicylic acid (mesalazine), and amorfrutins in human diseases has been well studied in vitro. However, their clinical significance in different diseases is largely unknown. Based on recent studies, five natural salicylates, including amorfrutin, ginkgolic acid, grifolic acid, tetrahydrocannabinolic acid, and cannabidiolic acid, showed potential roles in different challenging human diseases. This review summarizes together some of the recent information on multitarget regulatory activities of these natural salicylates and their pharmacological roles in human health.

## 1. Introduction

Traditionally, plants with a high level of salicylates have been used therapeutically throughout the world. Nowadays, salicylates containing plants are used as substitutes for nonsteroidal anti-inflammatory drugs (NSAIDs). In the fourth century B.C., the father of medicine Hippocrates reportedly prescribed extracts of willow leaves or barks to reduce fever and pain during childbirth [1]. In 1828, the active ingredient salicin (SA derivative) of the willow tree was isolated which is known to be converted to SA upon ingestion. Another natural salicylate methyl salicylate (MeSA), which is found in wintergreen, birch tree, mango, meadowsweet, guelder-rose, is used as analgesic medicine (for joint and muscular pain) and fragrance [2,3,4,5,6]. In the middle of the nineteenth century, an intense increase in the medicinal use of SA occurred due to the identification of salicin from willow plants as an anti-inflammatory compound. For growing demand, synthetic SA production was begun commercially in 1874. The most commonly used and recognized salicylate is aspirin (acetyl SA), which was first synthesized by Bayer in 1897. SA had some negative side effects, such as stomach irritation and bleeding, thus it was replaced with aspirin with fewer side effects [7]. Recent studies suggest that Aspirin not only reduces fever, inflammation, and pain but also reduces the risk of stroke, heart attack, and some type of cancers [8,9,10]. The mechanism of analgesia occurred by aspirin involves the suppression of prostaglandin synthesis by irreversible inhibition of the cyclooxygenases COX1 and COX2 [11]. Aspirin is also known to target other human proteins, such as nuclear factor-kappaB (NF-κB), cathepsin A (CTSA), ribosomal S6 kinase 2 (RSK2), and cyclin-dependent kinase 2 (CDK2) [12,13,14]. Both natural and synthetic salicylates have beneficial effects for the treatments of different diseases as salicylates can target different human proteins (Table 1). For example, 5-aminosalicylic acid targets arachidonate 5-lipoxygenase (ALOX5) and glyceraldehyde 3-phosphate dehydrogenase (GAPDH) protein [15,16]; Sulfasalazine targets ALOX5 and tumor necrosis factor (TNFα) protein [15,17]; Sodium salicylate targets COX2 and NF-κB [18]; GAPDH and High mobility group box 1 (HMGB1) both are targeted by acetyl 3-aminoethyl salicylic acid [16,19]. Thus, salicylates play an important role as a pharmacological agent in human health.

**Figure 1:**
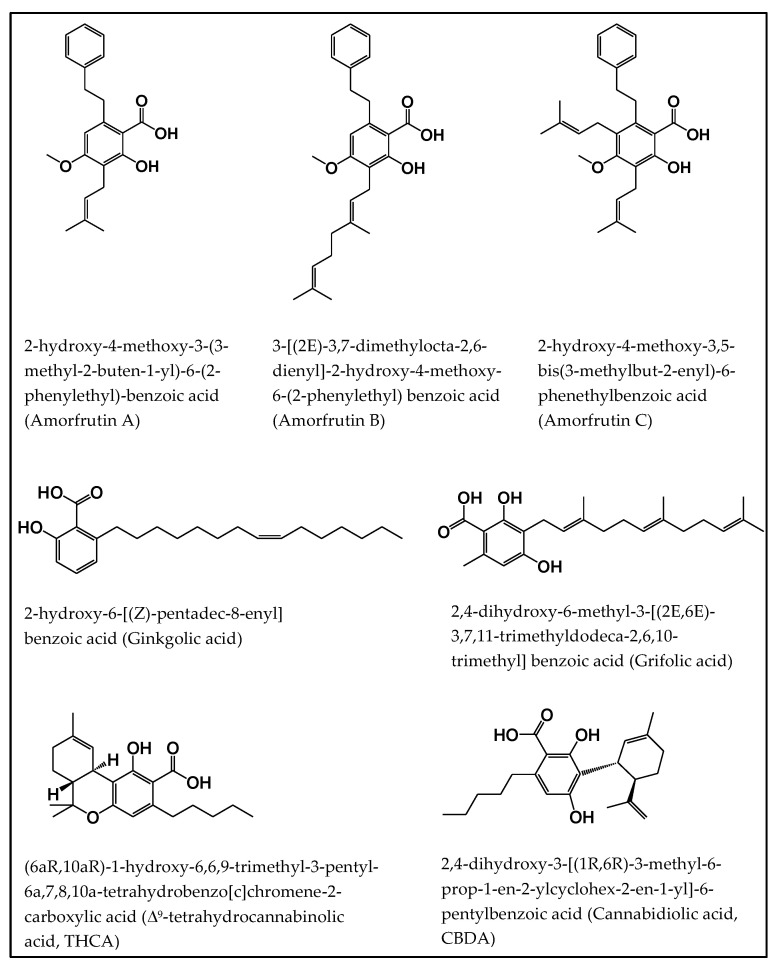
Chemical structures of our focused natural salicylates. Distinct phenyl moieties are contained in amorfrutins (A, B and C), THCA and CBDA, whereas fatty acids-based residues are contained in amorfrutins (A, B and C), gingkolic acid, grifolic acids, THCA and CBDA.

In this review, we focus on some natural salicylates, amorfrutin, ginkgolic acid, grifolic acid, tetrahydrocannabinolic acid, and cannabidiolic acid and discuss their multitarget inhibitory activities and pharmacological role in human disease (Figure 1). Amorfrutins are natural salicylates isolated from *Amorpha fruticosa* [25] and *Glycyrrhiza foetida* [26]. Different types of amorfrutins (A-D) are identified [27]. These amorfrutins are involved in many biological activities thus increasing the interest on it. The phenolic compound ginkgolic acids are found in leaves and fruits of *Ginkgo biloba* [28]. These are chemically 2-hydroxy-6-alkylbenzoic acids. Different ginkgolic acids structure such as C13:0, C15:1, and C17:1 have been designated according to their alkyl chain carbon number [29]. Recent studies suggest that ginkgolic acids have an anticancer property and some other pharmacological role so this compound may be a good target for drug development in human disease. The natural salicylate grifolic acid is isolated from the fruiting bodies of *Albatrellus confluens* fungus and this compound is a derivative of grifolin [30]. Grifolin shows anticancer activity in different types of cancer by inducing apoptosis and arrest of cell growth [31,32,33,34,35]. Some research suggests that grifolic acid also has anticancer activity [30,36]. *Cannabis sativa* L. is a medicinal plant that is rich in cannabinoids [37]. Δ^9^-tetrahydrocannabinolic acid (Δ^9^-THCA) and cannabidiolic acid (CBDA) are the most abundant cannabinoids in *C. sativa* L. and these are the acidic form of Δ^9^-THC and CBD respectively [38,39]. Besides the popular cannabinoids (THC and CBD), recently the other cannabinoids like Δ^9^-THCA and CBDA are gaining attention and interest from researchers due to their biological activities.

## 2. Pharmacological Roles of Natural Salicylates with Targets

### 2.1. Amorfrutins

Recent studies indicate that amorfrutins show various pharmacological effects including antidiabetic [40,41], anticancer [28], and anti-inflammatory [42] activities (Table 2).

#### 2.1.1. Antidiabetic Property

Type-2 diabetes is a worldwide increasing metabolic disease. For its prevention and treatment, new strategies are needed to develop. The gene expression of metabolism, inflammation, and other pathways in adipocytes is mainly regulated by the nuclear receptor PPARγ (peroxisome proliferator-activated receptor gamma) [73]. Weidner et al. suggest that amorfrutins perform as selective PPARγ modulators (SPPARγMs) which increase favorable changes in glucose metabolism and lipid profiles. Amorfrutins were isolated from the edible roots of licorice *G. foetida* and from the fruit of another legume *A. fruticosa* which is used as an ingredient in some condiments. In a competitive time-resolved fluorescent resonance energy transfer (TR-FRET) assay, the four amorfrutins showed the distinct Ki values selectivity for PPARγ subtypes (range from 236 to 354 nM), compared to other PPAR subtypes. Amorfrutins can regulate PPARγ gene expression selectively in adipocytes. Evaluation of in vivo effects in Type 2 diabetes mouse model suggested that amorfrutins act as antidiabetic agent [40]. Amorfrutin A and B inhibit insulin resistance formation, dyslipidemia, and liver steatosis through activation of PPARγ via ser273 phosphorylation [40,41,43]. These experiments together suggest that selective PPARγ-activation by dietary amorfrutins can develop a promising approach to fight against type-2 diabetes.

#### 2.1.2. Anticancer Activity

Today, cancer is a global concern, because of its leading position of causing death with about 8.8 million deaths and 18 million new cases per year [74,75]. Many different chemotherapeutic treatments are used for cancer, but those have some toxic side effects [76], so it is urgent to develop new promising anticancer agents. As an alternative approach, the clinical use of natural products or their synthetic analogs is considered to develop anticancer agents [77,78]. A recent study on amorfrutin C shows that it acts as an anticancer agent by inducing apoptosis and inhibiting proliferation of different cancer cell lines, including human colorectal cancer cells (HT-29 and T84), prostate cancer (PC-3), and breast cancer (MCF7) cells (IC_50_ values ranging from 8 to 16 μM in these cancer cell lines) through targeting mitochondria. Treatment of HT-29 cells with amorfrutin C induced apoptotic cell death accompanied by the formation of reactive oxygen species, DNA fragmentation, caspase activation, phosphatidylserine externalization, and poly (ADP-ribose) polymerase (PARP) cleavage. Synergistic apoptosis induction of amorfrutin C with alpha Fas receptor ligand (*α*FAS) and TNF-related apoptosis inducing ligand (TRAIL) in HT-29 cells suggest the stimulation of death receptor signaling pathway [28]. Taken together, amorfrutin C represents a promising candidate for anticancer drug development; however, its cellular target is not clearly identified.

#### 2.1.3. Anti-Inflammatory Effect

Although amorfrutin’s target PPARγ is the key regulator in metabolic regulation, it can inhibit inflammatory gene expression by a different mechanism including (i) direct interaction with transcription factor NF-κB, (ii) regulation of mitogen-activated protein kinase (MAPK) pathway by reducing p38 activation [79], (iii) suppression of inflammatory genes expression via SUMOylation-dependent pathway [80], and (iv) activating E3 ubiquitin ligase activity of PPARγ [81]. So, for the treatment of inflammatory disease, many clinical studies have been developed to evaluate the anti-inflammatory activity of PPARγ ligands. Amorfrutin A treatment in TNF-α- stimulated colon cells resulted in lower expression of various proinflammatory genes such as *COX-2*, *GRO-α*, *IL-8*, and *MIP-3α* suggesting that amorfrutins may have a beneficial effect for inflammatory disease, such as ulcerative colitis, via targeting PPARγ [42].

### 2.2. Ginkgolic Acids

Some recent studies suggest that ginkgolic acid (GA) may have a good candidate for developing a drug in different human diseases targeting various proteins. Its pharmacological role includes anticancer activity [44,45,46,47,48,49,50,51,82,83], neuroprotective role [52,53], and antimicrobial activity [54,55,84] (Table 2).

#### 2.2.1. Anticancer Activity

GA inhibited the proliferation of human Hep-2 cancer cells with the IC_50_ value of 20 µM [82] and induced cell death in human hepatoblastoma HepG2 cells via inducing apoptosis, autophagy, and mitochondrial dysfunction [51]. Bcl-2 is an antiapoptotic protein located in the outer membrane of mitochondria and plays its role by inhibiting cytochrome release and also caspase-3 activity; on the other hand, the proapototic protein Bax helps to release cytochrome and stimulates caspase-mediated cell death. So, lowering the Bcl-2/Bax ratio can be a good target for anticancer drug development [85,86,87,88,89]. Human Hep-2 cells and Tac8113 cells (human tongue squamous carcinoma cell line) treated by GA showed that reduced the Bcl-2/Bax ratio, and enhanced caspase-3 activity. GA also inhibited the growth of Tac8113 cells in a time- and dose-dependent manner with the IC_50_ value of 40 µM [50]. Oncogenic transcription factor STAT3 (Signal transducer and activator of transcription 3) involves in the development of hematological malignancies in multiple myeloma. Phosphorylation of STAT3 stimulates different cancer related proteins, such as Bcl-xl, Bcl-2 (tumorigenesis), Cyclin D1 (proliferation), MMP-9 (invasion), and VEGF (angiogenesis) [90,91,92]. Different tyrosine kinase called janus-like kinase (JAK), including JAK1, JAK2, JAK3, and TYK2 positively regulates the STAT3 pathway [93,94]; on the other hand, different protein tyrosine phosphatases (PTPs), including PTEN and SHP-1, negatively regulate STAT3 activation [95,96]. Thus, recent studies have been focused on the suppression of STAT3 activity for cancer treatment. Ginkgolic acid C (GAC) 17:1 suppressed STAT3 phosphorylation in multiple myeloma U266 cells and significantly reduced cell proliferation in both U266 and MM.1S cells in a dose- and time-dependent manner. GAC 17:1 upregulated the expression of PTEN and SHP-1 in protein and mRNA level, whereas it down-regulated the expression of STAT3-regulated gene products, such as Bcl-2, Bcl-xL, survivin, IAP-1, COX-2, cyclin D1, VEGF, MMP-9, and MMP-2, in multiple myeloma cells [49]. In SW480 colon cancer cells, GA treatment inhibited proliferation, migration, and invasion by stimulating adenosine monophosphate activated protein kinase (AMPK) activation and decreasing the expression of invasion-associated proteins such as matrix metalloproteinase-2 (MMP-2), C-X-C chemokine receptor type 4 (CXCR4), and urinary-type plasminogen activator (uPA) [48]. In lung cancer cells, GA treatment ameliorated migration and invasion by inhibition of the PI3K/Akt/mTOR signaling pathway [46]. GA reduced the viability of pancreatic cancer cells Panc-1 and BxPC-3. Both in vitro and in vivo results suggested that GA prevented the de novo lipogenesis of pancreatic cancer cells by inducing the activation of AMPK signaling pathway and suppressed several key enzymes (e.g., acetyl-CoA carboxylase [ACC], fatty acid synthase [FASN]) expression involved in lipogenesis [47]. In MCF-7 and MDA-MB 231 breast cancer cells, GA treatment showed antimigratory effects and inhibited the sumoylation of NEMO leading to inhibition of IκBα degradation. Consequently, reduced activity of NF-κB leads to the downregulation of NF-κB target genes, uPA, plasminogen activator inhibitor-1 (PAI-1), CXCR4, and MMP-9 [45]. GA inhibited the proliferation of renal cell carcinoma (RCC) cell lines 786-O and A498 by inactivating the epidermal growth factor receptor (EGFR) signaling pathway with the downregulation of p-Akt and p-Erk expression. Thus, GA targets different signaling pathways, including STAT3 pathway, PI3K/Akt/mTOR signaling pathway, and EGFR signaling pathway and causes downregulation of their associated proteins [44]. Microsomal prostaglandin E2 synthase-1 (mPGES-1)-derived prostaglandin E_2_ (PGE_2_) and leukotrienes (LTs) are both crucial mediators in the development of inflammation-associated cancer. GA inhibited the activity of mPGES-1 (IC_50_ = 0.7 µM) and 5-lipoxygenase (IC_50_ = 0.2 µM), the key enzyme in LT biosynthesis [83]. So, GA can be an efficient target for developing cancer treatment.

#### 2.2.2. Neuroprotective Activity

The aberrant SUMOylation process has been involved in neurodegenerative diseases [97]. GA directly binds SUMO-activating enzyme (E1) and inhibits the formation of E1-SUMO intermediate [53]. Neurodegenerative disorder Alzheimer’s disease (AD) is identified by the continuous loss of neurons, deposition of insoluble aggregates of two proteins in the brain, amyloid-β (Aβ) and the microtubule-associated protein tau (MAPT). Synaptic impairment occurs in this disease affecting the hippocampus and entorhinal cortex brain areas thus, hampered cognitive process and memory formation [98,99]. GA enhanced long-term potentiation (LTP) in the hippocampus, restored Aβ-mediated paired-pulse ratio (PPR) alteration, and rescued Aβ-mediated change in excitatory neurotransmission. The neuroprotective role of GA against Aβ-induced synaptic deterioration representing an effective approach to AD treatment [52].

#### 2.2.3. Antiviral Activity

HIV-1 protease plays an important role in the HIV viral life cycle as it breaks down the newly synthesized polyproteins to create the mature proteins of an HIV virion. Thus, HIV protease inhibitors (PIs) are very effective antiviral drugs that can reduce the morbidity and mortality of AIDS patients, thus significantly prolong their life [100]. GA inhibited HIV protease activity in a concentration-dependent manner with the IC_50_ of fewer than 30 μg/mL in the cell-free system and inhibited HIV-1SF162 infection in human peripheral blood mononuclear cells (PBMCs) in a concentration-dependent manner (50 and 100 μg/mL) [54]. GA showed an inhibitory effect on the fusion of a variety of enveloped viruses, including Zika virus (ZIKV), Herpes simplex virus type 1 (HSV-1), human cytomegalovirus (HCMV), human immune deficiency virus (HIV), Ebola virus (EBOV), influenza A virus (IAV), and Epstein Barr virus (EBV) and also inhibited a nonenveloped human adenovirus. In the case of postinfection, GA inhibited HSV-1 and CMV replication targeting protein and DNA synthesis by a secondary mechanism [55]. GA targets HIV protease enzyme and viral polymerase gene. As a PI, GA can play an important role in antiviral drug development.

### 2.3. Grifolic Acids

Grifolic acid is isolated from the fruiting bodies of *A. confluens* fungus and this compound is a derivative of grifolin [30]. This grifolin has anticancer properties [31,32,33,34,35]. Its acidic form grifolic acid is shown to have anticancer activity [30,36] (Table 2).

#### Antitumor Activity

Grifolic acid treatment reduced cell viability on GH3 cells, the rat anterior pituitary adenoma cells, in a dose- and time-dependent manner (from 2.5 μM to 20 μM) and resulted in total cell death after 6h treatment with 20 μM. Mitochondrial membrane potential (MMP) production was significantly decreased by grifolic acid (20 μM) 5 min after incubation and caused the maximal effect in 20 min. Grifolic acid also significantly decreased the cellular ATP level in GH3 cells. Without G-protein coupled receptor 120 (GPR120) activity, grifolic acid reduced GH3 adenoma cell viability by blocking NADH production in mitochondria, thereby decreasing MMP and ATP production [36]. Another report showed almost the same mechanism of grifolic acid in the case of osteosarcoma cell death. Grifolic acid treatment reduced cell viability in a time- and dose-dependent manner on four osteosarcoma cell lines U-2 OS, MG-63, Saos-2, and 143B. Total cell death occurred 6 h after treatment with 30 μM grifolic acid. In the animal model, intratumoral injection of grifolic acid increased necrosis of human osteosarcoma xenograft in nude mice without any observable toxicity [30].

### 2.4. Tetrahydrocannabinolic Acid (THCA) and Cannabidiolic Acid (CBDA)

The acidic forms of THC and CBD are THCA and CBDA, respectively, and these are the most abundant cannabinoids in *C. sativa* L. [39]. Based on recent studies, two phytocannabinoids THCA and CBDA may be a good candidate for developing an efficient drug for different human diseases treatment. The pharmacological role of THCA including immunomodulatory effect [56], anti-inflammatory role [57,58], neuroprotective role [59,60], and antineoplastic activity [61,62].

Based on in silico analysis, the drug likeness score of CBDA predicted it as possible G protein-coupled receptors (GPCRs) ligands, ion channel modulators, kinase inhibitors, nuclear receptor ligands, and protease inhibitors with moderately active in all bioactive scores [101]. According to recent studies CBDA has anticancer activity [63,64,65,66], anti-inflammatory activity [67,68,69], antiemetic effect [70,71], and anticonvulsant effect [72]. Due to its pharmacological property (Table 2), it is getting more and more attention.

#### 2.4.1. Immunomodulatory Effect

The cannabinoid receptor CB1 and CB2 are involved in immunomodulating actions of cannabinoids [102,103,104,105]. Other studies suggest that metabolic pathways and noncannabinoid receptors are involved with immunomodulatory effect of cannabinoids [106,107,108]. Importantly, agonists of cannabinoid receptor have a psychotropic effect [107]. Therefore, there is an effort to identify the compounds which have therapeutic effects but that are not able to activate CB1 and CB2 to avoid the psychotropic effect. THCA showed their immunomodulatory effect through a different metabolic pathway, without activating CB1 and CB2 [56]. THCA targeted phosphatidyl choline specific phospholipase C (PC-PLC) enzymatic activity in phospholipids metabolism and inhibits proinflammatory cytokine tumor necrosis factor alpha (TNF-α) release from lipopolysaccharide (LPS)-activated U937 macrophages and peripheral blood macrophages in a dose-dependent manner with the EC_50_ value of approximately 50 µM.

#### 2.4.2. Anti-Inflammatory Role

Cyclooxygenase 1 (COX1) and cyclooxygenase 2 (COX2) are essential to produce prostaglandins which are important for the inflammatory reaction. THCA inhibited COX1 (IC_50_ of 1700 µM) and COX2 (IC_50_ of 630 µM) using an enzyme-based in vitro assay and human colon adenocarcinoma HT29 cell line. Prostaglandin production was also inhibited (10% inhibition, 62.5 µM) in HT29 cell line by THCA [57]. Another report showed that THCA inhibited COX2 expression in a dose-dependent manner in three colon cancer cell lines HCT116, HT29, and CaCO2. In addition, THCA inhibited Matrix metallopeptidase 9 (MMP-9) expression in colon cell lines indicating that THCA plays an efficient role against colon inflammation [58].

CBDA exerted anti-inflammatory activity in receptor level by stimulating vanilloid 1 and ankyrin 1 transient receptor potential (TRP) channels (TRPV1 and TRPA1, respectively) or by antagonizing the Transient Receptor Potential Cation Channel Subfamily M Member 8 (TRPM8) with IC_50_ value ranging 0.9–1.6 µM [67]. COX2 is mainly involved in an inflammatory response. CBDA acts as a selective inhibitor of COX2 with an IC_50_ value of approximately 2 µM [68]. Intraperitoneal administration of rodent with CBDA (10 μg/kg) 60 min prior to treatment with carrageenan produced anti-inflammatory effects in a dose-dependent manner but orally did not [69].

#### 2.4.3. Neuroprotective Role

Noncompetitive inhibitors of the NADH ubiquinone reductase (complex 1), such as 1-methyl-4-phenyl pyridinium (MPP^+^), are used as a model compound in dopaminergic neuronal degeneration to study Parkinson’s disease (PD). THCA reduced the degenerative effect of MPP^+^ in dopaminergic neurons and increased cell survival at the highest tested dose of 10 µM in mice mesencephalic cultures [59]. Another model compound 3-nitropropionic acid (3-NPA) is used in the research of huntingtin disease (HD) which acts as a complex II inhibitor of the mitochondrial respiratory chain, resulting in progressive loss of locomotor and striatal degeneration. THCA mitigated degenerative effects of 3-NPA, through a PPARγ-dependent pathway in N2a cell [60].

Phytocannabinoids canabigerol (CBG) and cannabidivarin (CBDV) are the most promising candidates as neuroprotectants, while Δ9-tetrahydrocannabivarin (Δ9-THCV), Δ9-THCA, cannabichromene (CBC), and cannabinol (CBN) have limited but encouraging data as neuroprotectants. However, little is known with neuroprotective potential of CBDA [109].

#### 2.4.4. Anticancer Activity

THCA inhibited cell proliferation in various prostate carcinoma cell (PCC) lines, including (i) androgen-receptor positive cells (LNCaP and 22RV1) with IC_50_ of 22.1 ± 2 µM or 17.1 ± 1 µM in the presence or absence of serum, respectively, (ii) androgen-receptor negative cells (DU-145 and PC-3) with IC_50_ of >25 µM (21.9% inhibition) or 21.6 ± 2 µM in the presence or absence of serum, respectively [62]. Cell proliferation of two different human breast carcinoma (HBC) cells, triple-negative MDA-MB-231 and HER2-negative MCF-7, were inhibited with IC_50_ value of 18.2 ± 5 µM and 9.8 ± 0.4 µM, respectively [61].

CBDA reduced COX2 expression in triple-negative MDA-MB-231 human breast cancer cell with a concentration of 5 µM by abrogating the transcriptional activities of both activator protein-I (AP-I) and peroxisome proliferator-activated receptor PPARβ/δ [63]. CBDA inhibited human breast cancer cell metastasis by suppressing COX2 and proto-oncogene c-Fos expression and upregulating the expression of SHARP1(1.72-fold), a suppressor of breast cancer metastasis [63,64,65,66].

#### 2.4.5. Antiemetic Effect and Anticonvulsant Effect

5-HT1A receptors control the antinausea effects of CBDA. In vivo mice study showed that CBDA inhibited vomiting caused by toxins and induced 5-HT1A receptor activity [70,71]. The pharmacokinetics of phytocannabinoid acids (including CBDA with others) showed that plasma level absorption occurred rapidly, and the brain/plasma ratio was very low. However, when CBDA was administered in an alternate Tween 80-based vehicle, the value of the brain/plasma ratio was 1.9 and in *Scn1a*^RX/+^ mouse model of Dravet syndrome, and CBDA showed potent anticonvulsant activity [72].

## 3. Conclusions

Plant derived natural compounds are used throughout the world for medicinal purposes. Plant derived salicylates target many human proteins and can play prominent roles in human disease treatment. Initially, researchers focused on acetyl salicylate (aspirin) and showed that it can be a good target for drug development for many human diseases. Recent findings suggest that many natural salicylates have pharmacological roles. Our focused salicylates amorfrutins, ginkgolic acid, grifolic acid, THCA, and CBDA target different human proteins and pathways. It appears clear that these natural salicylates may be promising natural compounds in treating different human diseases. Some medicinal plants and natural products showed relevant results in clinical trials [110,111,112]. However, there are very limited clinical trials available with these salicylates. Our review suggests that further clinical trials with different salicylates on different human diseases will be needed based on many preclinical results with amorfrutins (antidiabetic, anticancer, and anti-inflammatory activities), ginkgolic acid (anticancer, neuroprotective, and antiviral activities), grifolic acid (antitumor activity), THCA (immunomodulatory, anti-inflammatory, neuroprotective, and antineoplastic activities), and CBDA (anticancer, anti-inflammatory, antiemetic, and anticonvulsant activities).

## Figures and Tables

**Table 1:** Salicylates with their structures and targeted protein.

Salicylates & Structure	Proteins	References
Salicylic acid	Ferrochelatase (FECH)	[16]
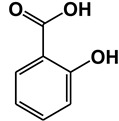	Cyclin-dependent kinase 2 (CDK2)	[14]
	α-Enolase (ENO1)	[20]
	Pyruvate kinase isozyme M2 (PKM2)	[20]
Acetylsalicylic acid (Aspirin)	Cyclooxygenase-1 (COX-1)	[21,22]
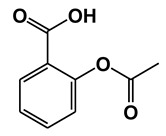	Cyclooxygenase-2 (COX-2)	[18,21,22]
	Nuclear factor-kappaB (NF-κB)	[11]
	Cathepsin A (CTSA)	[12]
	Inhibitor of nuclear factorkappa-B kinase subunit beta (Iκκ-β)	[23]
	Ribosomal S6 kinase 2 (RSK2)	[13]
	Cyclin-dependent kinase 2 (CDK2)	[14]
5-aminosalicylic acid (Mesalazine)	Arachidonate 5-lipoxygenase (ALOX5)	[15]
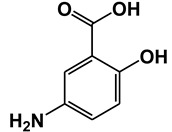	Glyceraldehyde 3-phosphate dehydrogenase (GAPDH)	[16]
2-(2-hydroxybenzoyl) oxybenzoic acid (Salsalate)	Acetyltransferase p300 (P300)	[24]
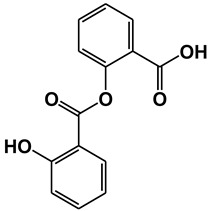		
Acetyl 3-aminoethyl salicylic acid (Ac3AESA)	Glyceraldehyde 3-phosphate dehydrogenase (GAPDH)	[16]
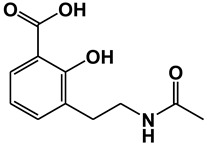	High mobility group box 1 (HMGB1)	[19]
2-hydroxy-5-[(E)-2-{4-[(pyridin-2-yl) sulfamoyl] phenyl} diazen-1-yl] benzoic acid (Sulfasalazine)	Tumor necrosis factor alpha (TNFα)	[17]
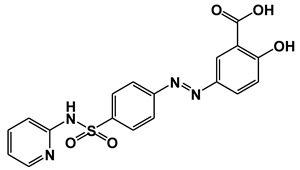	Arachidonate 5-lipoxygenase (ALOX5)	[15]
Sodium salicylate	Cyclooxygenase-2 (COX-2)	[18,21,22]
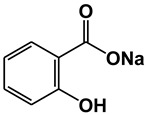	Nuclear factor-kappaB(NF-κB)	[11]
	Inhibitor of nuclear factorkappa-B kinase subunit beta (Iκκ-β)	[23]

**Table 2:** Source, biological activities, and mechanism of action of our focused natural salicylates.

Natural Salicylates	Source	Biological Activity	Mechanism of Action	Ref
Amorfrutins	*A. fruticosa* L. (bastard indigo), *G. foetida* Desf. (licorice)	Antidiabetic	Inhibition of insulin resistance formation, dyslipidemia, liver steatosis, and induction of PPARγ ser273 phosphorylation by HFD	[40,41,43]
		Anticancer	Formation of reactive oxygen species, DNA fragmentation, caspase activation, phosphatidylserine externalization, and PARP cleavage induced apoptosis in colorectal cancer cells	[28]
		Anti-inflammatory	Lowering the expression of various inflammatory genes such as COX-2, GRO-α, IL-8, and MIP-3α in TNF-α- stimulated colon cells	[42]
Ginkgolic acids	*G. biloba* L.	Anticancer	Inhibition of the proliferation of renal cell carcinoma (RCC) cell lines 786-O and A498 by inactivating epidermal growth factor receptor (EGFR) signaling pathway with the downregulation of p-Akt and p-Erk expression	[44]
			Inhibition of IκBα degradation and reduction of the activity of NF-κB in MCF-7 and MDA-MB 231 breast cancer cells	[45]
			Inhibition of the PI3K/Akt/mTOR signaling pathway of lung cancer cells	[46]
			Reduction of the cell viability and prevention of the de novo lipogenesis of pancreatic cancer cells	[47]
			Reduction of proliferation, migration, and invasion of SW480 colon cancer cells. Stimulation of AMPK activation and decreasing the expression of invasion-associated proteins, such as (MMP)-2, (CXCR4) and uPA	[48]
			Suppression of STAT3 phosphorylation and reduction of cell proliferation in multiple myeloma cancer cells. Upregulation of the expression of PTEN and SHP-1	[49]
			Inhibition of the growth of human tongue squamous carcinoma cells. Reduction of the Bcl-2/Bax ratio and stimulation of caspase-3 activity	[50]
			Inhibition the cell proliferation and induction of cell death through a combination of apoptosis, autophagy, and the mitochondrial pathway in human hepatoblastoma HepG2 cells	[51]
		Neuroprotective	Enhancement of long-term potentiation (LTP) in the hippocampus, restoring the Aβ-mediated paired-pulse ratio (PPR) alteration and rescuing the Aβ-mediated change in excitatory neurotransmission in mice model.	[52]
			Inhibition of the formation of E1-SUMO intermediate in an in situ cell-based SUMOylation assay	[53]
		Antiviral	Inhibition of HIV protease activity of HIV-1SF162 infection in human peripheral blood mononuclear cells	[54]
			The inhibitory effect on the fusion of a variety of enveloped viruses, including ZIKV, HSV-1, HCMV HIV, EBOV, IAV and EBV, and a nonenveloped human adenovirus	[55]
Grifolic acid	*A. confluens*	Antitumor	Reduction of cell viability by blocking NADH production and inhibiting MMP and ATP production on both GH3 cells and osteosarcoma cell	[30,36]
Tetrahydro-cannabinolic acid (THCA)	*C. sativa*	Immunomodulatory	Targeting PC-PLC enzymatic activity in phospholipids metabolism and inhibition of tumor TNF-a release from LPS-activated U937 macrophages and peripheral blood macrophages	[56]
		Anti-inflammatory	Inhibition COX1 and COX2 expression, Reduction of prostaglandin production also inhibition MMP9 expression in colon cancer cell	[57,58]
		Neuroprotective	Reduction of dopaminergic neurons degenerative effect of MPP+, increasing cell survival in mice mesencephalic cultures	[59]
			Reduction of degenerative effects of 3-NPA, through a PPARγ-dependent pathway in N2a cell and in vivo mice model	[60]
		Antineoplastic	Inhibition of cell proliferation in the breast cancer cell and prostate carcinoma cell	[61,62]
Cannabidiolic acid (CBDA)	*C. sativa*	Anticancer	Inhibition of the transcriptional activities of both activator protein I (AP-I) and peroxisome proliferator-activated receptor PPARβ/δ in breast cancer cell	[63]
			Inhibition of human breast cancer cell metastasis by suppressing COX2 and proto-oncogene c-Fos expression and upregulation of the expression of SHARP1	[64,65,66]
		Anti-inflammatory	Stimulation of vanilloid 1 and ankyrin 1 transient receptor potential (TRP) channels (TRPV1 and TRPA1, respectively), and antagonize a receptor, the Transient Receptor Potential Cation Channel Subfamily M Member 8 (TRPM8) in HEK 293 cells	[67]
			Selective inhibition of COX2. Reduction of inflammation when intraperitoneally administered in a rodent model of carrageenan-induced acute inflammation in the rat hind paw.	[68,69]
		Antiemetic	Induction 5-HT1A receptors activity in vivo in mice	[70,71]
		Anticonvulsant	Increasing the temperature threshold at which the Scn1aRX/+ mice had a generalized tonic-clonic seizure.	[72]
